# Supplementary material for: Results of an early second PCR test performed on SARS-CoV-2 positive patients may support risk assessment for severe COVID-19
Source: Sci Rep. 2021 Oct 14;11:20463. doi: 10.1038/s41598-021-99671-5 (PMC8516879; doi:10.1038/s41598-021-99671-5)
Supplement: Supplementary file 1 — Supplementary Information. [file 41598_2021_99671_MOESM1_ESM.docx]

**Supplementary Info**

**Supplementary Table 1**

Probability analysis for deterioration to severe condition after receiving test result of a 2nd PCR test done within a week following COVID-19 positive diagnosis

| **Age (yrs)** | **Test results** | **# of patients** | **# of patients with severe condition** | **Probability to develop severe condition** | **95% CI** |
| --- | --- | --- | --- | --- | --- |
| 18-59 | Positive | 624 | 22 | 3.53% | [2.08%-4.97%] |
|  | Negative | 663 | 6 | 0.90% | [0.18%-1.63%] |
|  | Not tested | 12191 | 106 | 0.87% | [0.70%-1.03%] |
| 60-79 | Positive | 158 | 31 | 19.62% | [13.43%-25.81%] |
|  | Negative | 108 | 6 | 5.56% | [1.24%-9.88%] |
|  | Not tested | 1673 | 121 | 7.23% | [5.99%-8.47%] |
| ≥80 | Positive | 91 | 38 | 41.76% | [31.63%-51.89%] |
|  | Negative | 39 | 5 | 12.82% | [2.33%-23.31%] |
|  | Not tested | 275 | 81 | 29.45% | [24.07%-34.84%] |
| All | Positive | 873 | 91 | 10.42% | [8.40%-12.45%] |
|  | Negative | 810 | 17 | 2.10% | [1.11%-3.09%] |
|  | Not tested | 14139 | 308 | 2.18% | [1.94%-2.42%] |

**Supplementary Table 2**

Statistical evaluation of the severe cases following 2^nd^ positive or negative PCR test results in different age groups

| **Age (yrs)** | **# of patients with 2^nd^ positive test** | **# of patients with severe condition and 2^nd^ positive test** | **# of patients with 2^nd^ negative test** | **# of patients with severe condition and 2^nd^ negative test** | **p-value** |
| --- | --- | --- | --- | --- | --- |
| 18-59 | 624 | 22 | 663 | 6 | 0.0019 |
| 60-79 | 158 | 31 | 108 | 6 | 0.0013 |
| ≥80 | 91 | 38 | 39 | 5 | 0.0016 |
| All | 873 | 91 | 810 | 17 | <0.0001 |

**Supplementary Table 3**

Statistical comparison of the severe cases following 2^nd^ PCR test results between the different age groups

| **Test Result** | **Age group1** | **Age group2** | **# of patients group 1** | **# of patients with severe condition Group1** | **# of patients group 2** | **# of patients with severe condition Group 2** | **p-value** |
| --- | --- | --- | --- | --- | --- | --- | --- |
| Positive | 18-59 | 60-79 | 624 | 22 | 158 | 31 | <0.001 |
|  | 60-79 | >=80 | 158 | 31 | 91 | 38 | <0.001 |
| negative | 18-59 | 60-79 | 663 | 6 | 108 | 6 | 0.007 |
|  | 60-79 | >=80 | 108 | 6 | 39 | 5 | 0.281 |
| no test | 18-59 | 60-79 | 12191 | 106 | 1673 | 121 | <0.001 |
|  | 60-79 | >=80 | 1673 | 121 | 275 | 81 | <0.001 |

**Supplementary Table 4**

Odds ratio (OR) analysis of a second positive SARS-CoV-2 PCR test result within a week of COVID-19 diagnosis and deterioration to severe COVID-19.

| **Age (yrs)** | **OR** | **95%CI** | **p-value** |
| --- | --- | --- | --- |
| 18-59 | 3.94 | [1.59-9.79] | 0.0031 |
| 60-79 | 4.31 | [1.72-10.78] | 0.0018 |
| ≥80 | 5.36 | [1.90-15.16] | 0.0015 |

**Supplementary Table 5**

Probability analysis for deterioration to severe condition after receiving test result of a 2nd PCR test done within a week following COVID-19 positive diagnosis in hospital and community settings

| **Age (yrs)** | **Test within a week of diagnosis** | **# of patients** | **# of patients with severe condition** | **Probability to develop severe condition** | **95% CI** |
| --- | --- | --- | --- | --- | --- |
| Admitted to hospital >18 yrs | Positive test | 304 | 77 | 25.33% | [20.44%-30.22%] |
|  | Negative test | 113 | 16 | 14.16% | [7.73%-20.59%] |
|  | Performed a second test | 417 | 93 | 22.30% | [18.31%-26.30%] |
| In the community >18 yrs | Positive test | 569 | 14 | 2.46% | [1.19%-3.73%] |
|  | Negative test | 697 | 1 | 0.14% | [0.00%-0.42%] |
|  | Performed a second test | 1266 | 15 | 1.18% | [0.59%-1.78%] |
| >18 yrs | Positive test | 873 | 91 | 10.42% | [8.40%-12.45%] |
|  | Negative test | 810 | 17 | 2.10% | [1.11%-3.09%] |
|  | Performed a second test | 1683 | 108 | 6.42% | [5.25%-7.59%] |
| Admitted to hospital >60 yrs | Positive test | 145 | 57 | 39.31% | [31.36%-47.26%] |
|  | Negative test | 47 | 10 | 21.28% | [9.58%-32.98%] |
|  | Performed a second test | 192 | 67 | 34.90% | [29.09%-40.70%] |
| In the community >60 yrs | Positive test | 104 | 12 | 11.54% | [5.40%-17.68%] |
|  | Negative test | 100 | 1 | 1.00% | [0.00%-2.95%] |
|  | Performed a second test | 204 | 13 | 6.37% | [3.12%-9.62%] |
| >60 yrs | Positive test | 249 | 69 | 27.71% | [22.15%-33.27%] |
|  | Negative test | 147 | 11 | 7.48% | [3.23%-11.74%] |
|  | Performed a second test | 396 | 80 | 20.20% | [16.60%-23.81%] |

**Supplementary Table 6**

Statistical evaluation of the severe cases following a 2^nd^ positive or negative PCR test results in different clinical settings

| **Age (yrs)** | **# of patients with 2^nd^ positive test** | **# of patients with severe condition and 2^nd^ positive test** | **# of patients with 2^nd^ negative test** | **# of patients with severe condition and 2^nd^ negative test** | **p-value** |
| --- | --- | --- | --- | --- | --- |
| Admitted to hospital >18 yrs | 304 | 77 | 113 | 16 | 0.0174 |
| In the community >18 yrs | 569 | 14 | 697 | 1 | 0.0002 |
| >18 yrs | 873 | 91 | 810 | 17 | <0.0001 |
| Admitted to hospital >60 yrs | 145 | 57 | 47 | 10 | 0.0330 |
| In the community >60 yrs | 104 | 12 | 100 | 1 | 0.0030 |
| >60 yrs | 249 | 69 | 147 | 11 | <0.0001 |

**Supplementary Table 7**

Odds ratio (OR) analysis of a second positive SARS-CoV-2 PCR test result within a week of COVID-19 diagnosis and deterioration to severe COVID-19 in hospital and community settings.

| **Age (yrs) -** | **Odds ratio** | **95%CI** | **p-value** |
| --- | --- | --- | --- |
| Admitted to hospital >=18 yrs | 1.91 | [1.02-3.57] | 0.0418 |
| In the community >=18 yrs | 13.49 | [1.72-105.56] | 0.0132 |
| All adults >=18 yrs | 4.35 | [2.52-7.53] | <0.0001 |
| Admitted to hospital >=60 yrs | 2.31 | [1.03-5.15] | 0.041 |
| In the community >=60 yrs | 10.47 | [1.31-83.67] | 0.027 |
| All adults >=60 yrs | 4.42 | [2.23-8.76] | <0.0001 |

**Supplementary Table 8**

The probability for a negative test result relative to the date of diagnosis in patients with a “positive window”.

| **Day from diagnosis** | **# of positive test results** | **# of negative test result** | **Total # of tests** | **Probability for a negative test** |
| --- | --- | --- | --- | --- |
| 1 | 112 | 5 | 117 | 4.27% |
| 2 | 77 | 8 | 85 | 9.41% |
| 3 | 104 | 11 | 115 | 9.57% |
| 4 | 99 | 20 | 119 | 16.81% |
| 5 | 89 | 30 | 119 | 25.21% |
| 6 | 128 | 33 | 161 | 20.50% |
| 7 | 185 | 42 | 227 | 18.50% |
| 8 | 164 | 52 | 216 | 24.07% |
| 9 | 171 | 73 | 244 | 29.92% |
| 10 | 206 | 84 | 290 | 28.97% |
| 11 | 258 | 115 | 373 | 30.83% |
| 12 | 604 | 307 | 911 | 33.70% |
| 13 | 390 | 192 | 582 | 32.99% |
| 14 | 251 | 132 | 383 | 34.46% |
| 15 | 206 | 129 | 335 | 38.51% |
| 16 | 179 | 92 | 271 | 33.95% |
| 17 | 177 | 84 | 261 | 32.18% |
| 18 | 146 | 83 | 229 | 36.24% |
| 19 | 201 | 121 | 322 | 37.58% |
| 20 | 165 | 105 | 270 | 38.89% |
| 21 | 112 | 81 | 193 | 41.97% |

**Supplementary Table 9**

Probability analysis for deterioration to severe condition following a 2nd PCR test done within a week following COVID-19 positive diagnosis for patients with and without “positive window”.

|  | **Test result** | **# of patients** | **# of patients with severe condition** | **Probability to develop severe condition** | **95% CI** |
| --- | --- | --- | --- | --- | --- |
| Positive diagnosis >=18 | Positive | 873 | 91 | 10.42% | [8.40%-12.45%] |
|  | Negative | 810 | 17 | 2.10% | [1.11%-3.09%] |
|  | No test | 14139 | 308 | 2.18% | [1.94%-2.42%] |
| Positive window >=18 | Positive | 563 | 59 | 10.48% | [7.95%-13.01%] |
|  | Negative | 124 | 4 | 3.23% | [1.20%-6.34%] |
|  | No test | 4622 | 140 | 3.03% | [2.53%-3.52%] |
| No Positive window >=18 | Positive | 310 | 32 | 10.32% | [6.94%13.71%] |
|  | Negative | 686 | 13 | 1.90% | [0.87%-2.92%] |
|  | No test | 9517 | 168 | 1.77% | [1.50%-2.03%] |
| Positive diagnosis >=60 | Positive | 249 | 69 | 27.71% | [22.15%-33.27%] |
|  | Negative | 147 | 11 | 7.48% | [3.23%-11.74%] |
|  | No test | 1948 | 202 | 10.37% | [9.02%-11.72%] |
| Positive window >=60 | Positive | 158 | 42 | 26.58% | [19.69%-33.47%] |
|  | Negative | 24 | 2 | 8.33% | [0.00%-19.39%] |
|  | No test | 783 | 86 | 10.98% | [8.79%-13.17%] |
| No Positive window >=60 | Positive | 91 | 27 | 29.67% | [21.43%-37.91%] |
|  | Negative | 123 | 9 | 7.32% | [2.87%-11.76%] |
|  | No test | 1165 | 116 | 9.96% | [8.24%-11.68%] |

**Supplementary Table 10**

Statistical evaluation of the severe cases following a 2^nd^ positive or negative PCR test results for patients with or without a “positive window”

| **Age (yrs)** | **# of patients with 2^nd^ positive test** | **# of patients with severe condition and 2^nd^ positive test** | **# of patients with 2^nd^ negative test** | **# of patients with severe condition and 2^nd^ negative test** | **p-value** |
| --- | --- | --- | --- | --- | --- |
| Positive diagnosis >=18 yrs | 873 | 91 | 810 | 17 | <0.0001 |
| Positive window >=18 yrs | 563 | 59 | 124 | 4 | 0.0092 |
| No Positive window >=18 yrs | 310 | 32 | 686 | 13 | <0.0001 |
| Positive diagnosis >=60 yrs | 249 | 69 | 147 | 11 | <0.0001 |
| Positive window >=60 yrs | 158 | 42 | 24 | 2 | 0.0680 |
| No Positive window >=60 yrs | 91 | 27 | 123 | 9 | <0.0001 |

**Supplementary Table 11**

Odds ratio (OR) analysis of a second positive SARS-CoV-2 PCR test result within a week of COVID-19 diagnosis and deterioration to severe COVID-19 for patients with and without “positive window”.

| **Group** | **Odds ratio** | **95%CI** | **p-value** |
| --- | --- | --- | --- |
| Positive window >=18 | 2.87 | [0.99-8.32] | 0.05 |
| No Positive window >=18 | 4.80 | [2.37-9.72] | <0.0001 |
| Positive diagnosis >=18 | 4.35 | [2.52-7.53] | <0.0001 |

| **Group** | **Odds ratio** | **95%CI** | **p-value** |
| --- | --- | --- | --- |
| Positive window >=60 | 3.51 | [0.78-15.81] | 0.102 |
| No Positive window >=60 | 5.28 | [2.28-12.22] | 0.0001 |
| Positive diagnosis >=60 | 4.42 | [2.23-8.76] | <0.0001 |

**Supplementary Table 12**

Probability analysis for deterioration to severe condition after receiving test result of a 2nd PCR test done between days 1 and 7 following COVID-19 positive diagnosis.

| **Time following diagnosis (d)** | **Test result** | **# of patients** | **# of patients with severe condition** | **Probability to develop severe condition** | **p-value** |
| --- | --- | --- | --- | --- | --- |
| **d1** | Positive | 199 | 16 | 8.04% |  |
|  | Negative | 92 | 6 | 6.52% | 0.42 |
| **d2** | Positive | 115 | 15 | 13.04% |  |
|  | Negative | 123 | 3 | 2.44% | 0.002 |
| **d3** | Positive | 143 | 14 | 9.79% |  |
|  | Negative | 134 | 1 | 0.75% | 0.001 |
| **d4** | Positive | 116 | 14 | 12.07% |  |
|  | Negative | 119 | 1 | 0.84% | <0.001 |
| **d5** | Positive | 101 | 7 | 6.93% |  |
|  | Negative | 114 | 2 | 1.75% | 0.06 |
| **d6** | Positive | 155 | 15 | 9.68% |  |
|  | Negative | 116 | 0 | 0.00% | <0.001 |
| **d7** | Positive | 209 | 24 | 11.48% |  |
|  | Negative | 156 | 5 | 3.21% | 0.003 |
